# Creb3l1 Overexpression Improves Flexor Tendon Repair in Aged Rats

**DOI:** 10.1111/acel.70682

**Published:** 2026-08-23

**Authors:** Qian Qian Yang, Zhanghao Huang, Yan Xue, Jia Yu Shi, You Lang Zhou

**Affiliations:** ^1^ Hand Surgery Research Center, Research Center of Clinical Medicine Affiliated Hospital of Nantong University, Medical School of Nantong University Nantong P.R. China; ^2^ Thoracic Surgery Affiliated Hospital of Nantong University Nantong P.R. China

**Keywords:** aging, Creb3l1, gliding function, strength, tendon injury

## Abstract

Aging increases tendon injury incidence and poor repair outcomes, but age‐related differences in tendon mechanical properties and underlying mechanisms remain unclear. We first analyzed data from the Global Burden of Disease (GBD) database to characterize the associations between aging and population‐level changes in tendon injury burden. We found the age‐standardized incidence rate (ASIR) of tendon injuries caused by mechanical force decreased with advancing age, whereas the age‐standardized prevalence rate (ASPR) increased with age, and the years lived with disability (YLDs) due to tendon injuries also showed an increasing trend with age. Guided by these epidemiological observations, we further performed ex vivo biomechanical assessments using aged rat flexor digitorum longus (FDL) tendon injury models to validate age‐related mechanical deterioration at the tissue level, revealing significantly compromised mechanical properties in aged female rat tendons. To uncover the molecular drivers underlying such age‐dependent mechanical degeneration, we conducted single‐cell RNA sequencing of tendon tissues and identified downregulated expression of *Col1a1* and *Sparc* in aged tendons. According to the JASPAR website prediction, the transcription factor Creb3l1 could bind to the promoter regions of both *Col1a1* and *Sparc* genes simultaneously, and its overexpression promoted their expression. Finally, in vivo Creb3l1 overexpression upregulated the levels of Col1a1 and Sparc proteins and improved the gliding function, healing strength, and elastic modulus of aged tendons, especially in aged male rats. Collectively, this study links population‐based epidemiological evidence with preclinical functional and mechanistic validation, providing insights for risk prediction and novel therapeutic targets for age‐related tendon injuries.

## Graphical abstract

Aging impairs flexor tendon repair, with aged tendons showing reduced Col1a1 and Sparc expression and compromised mechanical properties. The transcription factor Creb3l1 binds both gene promoters and drives their expression; its overexpression restores Col1a1 and Sparc levels and improves the gliding function, healing strength, and elastic modulus of injured aged rat tendons.

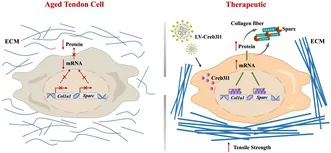

## 1 Introduction

Tendon injury can be acute or chronic, caused by internal or external factors alone or in combination (Sharma and Maffulli 2005). With the population rapidly aging, the incidence of sports injuries in old age remains at a relatively high level (Kammerlander et al. 2012). An epidemiological survey revealed that the age distribution of primary Achilles tendon rupture and re‐rupture showed a bimodal pattern, reaching its peak in the fifth decade and a secondary peak in the older population (Maempel et al. 2022). Another survey found that rotator cuff tears and biceps tendon ruptures are more common in older adults than the younger (Clayton and Court‐Brown 2008). For flexor tendon injury, older patients were likely to have a slower healing process and impaired digital range of motion during the first year after surgery (Edsfeldt et al. 2019).

Some researchers have investigated the functional changes of flexor tendons with aging using mouse tendon models. Zuskov et al. subjected young and aged mouse patellar tendons and flexor digitorum longus tendons to fatigue loading and found that the crimp pattern of Type I collagen in tendons undergoes permanent alterations with aging and fatigue loading (Zuskov et al. 2020). Connizzo et al. cultured flexor tendon explants from young and aged male and female mice under stress‐deprived ex vivo conditions and demonstrated that aging markedly impairs the tendon response to mechanical stress deprivation, with distinct sex‐dependent differences in the aging response (Connizzo et al. 2020). Aggouras et al. treated young and aged mouse flexor tendon explants with different magnitudes of cyclic tensile strain and revealed that aged tendons exhibit a blunted response to mechanical strain and reduced mechanical adaptability (Aggouras et al. 2024). Ackerman et al. examined mechanical and cellular characteristics of three tendon types in male and female mice across multiple ages and reported that aging does not substantially alter the ultimate load of intact tendons, while injured tendons from aged mice display significantly inferior mechanical healing outcomes (Ackerman et al. 2017). Collectively, these mouse‐based studies consistently demonstrate that aging impairs the mechanical adaptability, stress responsiveness, and healing capacity of flexor tendons. However, the biological mechanisms underlying age‐related deterioration of tendon mechanical properties remain unclear to date.

In general, the aging of tendons is related to cellular aging. Kohler et al. explored the transcriptome changes of human TSPC with age and found that genes with significant changes in mRNA expression levels are related to cell communication, cytoskeleton, and actin dynamics, and motility migration (Kohler et al. 2013). Peffers et al. also demonstrated that the transcriptome of a normal human Achilles tendon dynamically changes with age (Peffers et al. 2015). Animal studies have shown that aging leads to a decrease in tendon cell density in rabbits and rats, and the content of elastin decreases significantly in horses (Godinho et al. 2017; Nagy et al. 1969; Nakagawa et al. 1996). Moreover, tendon cells also exhibit poor proliferation and migration abilities (Ross et al. 2011; Zhou et al. 2010). Therefore, aging can lead to changes in cellular function in tendon tissue, thereby altering the ability to respond to mechanical force signals.

In order to further clarify the weakening of tendon function caused by aging, in this study, we would first reveal the correlation between age and tendon injury from the perspectives of epidemiology and experimental animals. Then we used single‐cell sequencing technology (scRNA‐seq) to clarify the effects of aging on various cells in tendons to accurately explain how aging alters the mechanical properties of tendons. Finally, we improved the poor repair outcome of injured aged tendons by regulating the identified differential genes. The results of this study contribute to understanding the characteristics of tendon mechanical properties after tendon injury in aged individuals of different genders and provide a cellular level explanation for this phenomenon.

## 2 Methods

### 2.1 Data Sources

The GBD data used in this study include information on 371 diseases and injuries from 1990 to 2021, covering more than 204 countries and regions (http://ghdx.healthdata.org/gbd‐results‐tool). From the GBD database, we extracted, for global and its regions, the number of incident cases, prevalent cases related to tendon injuries due to mechanical force exposure, as well as the corresponding age‐standardized incidence and prevalence by sex and age group. Individuals aged 0–14 years were grouped together, and from age 15 years onward, each 5‐year increment formed one age group, yielding a total of 18 age groups (Zhou et al. 2025).

### 2.2 Evaluation Indicators

To gain a deeper understanding of the data's changing patterns, we categorized patient numbers into aging, epidemiological changes, and population. The Socio‐Demographic Index (SDI) measures regional development levels based on a combination of factors, such as total fertility rate for individuals aged 15 and above, per capita income distribution, and years of education for those aged 15 and older. The SDI ranges from 0 to 1, and regions are classified into low, medium, or high SDI categories according to their SDI scores (Yuan et al. 2025).

### 2.3 Age–Period–Cohort (APC) Model

The age‐period‐cohort model is used to analyze time‐series data and to understand how different time‐related factors influence various outcomes. The main analysis examines three different factors influencing a specific outcome or trend: age effect, period effect, and cohort effect. The age effect refers to changes in the outcome of interest resulting from biological and social processes associated with an individual's aging. The period effect arises when certain factors exert an influence at a given point in time, affecting all age groups simultaneously. The cohort effect stems from the unique experiences shared by individuals born within the same time frame as they progress through life (Tan et al. 2025).

### 2.4 Animal Preparation and Groups

The animal experimental procedures were approved by the Animal Care Committee of the Experimental Animal Center of Nantong University (No. S20230112‐020). We defined 18‐month‐old Sprague‐Dawley (SD) rats as “aged rats” and 8‐week‐old SD rats as “young rats”. Total 40 tendons from 40 rats (10 aged male rats, 10 aged female rats, 10 young male rats, 10 young female rats) were subjected to mechanical property measurement, such as gap, Young's modulus, and maximum tension measurements.

### 2.5 Mechanical Property Measurement

To prevent water loss, we harvested every 5 tendons for one test to ensure all tendons complete the tests within half an hour after detachment. In detail, after euthanizing the rats, FDL tendons were cut from the muscle‐tendon junction to the site of the proximal phalanx and measured for length and weight. All tendons were firstly subjected to 100 stretching cycles from 1 to 10 N at a rate of 6 mm/s by a tensile testing machine (Instron 3365, Instron Corp, Norwood, MA, USA). Then we performed a complete transverse cut at the center position of the tendons and repaired with a 2‐strand Kessler's method by 5–0 suture (Prolene; Ethicon, Somerville, NJ, USA). The tendons were mounted to the tensile testing machine again and pulled at a rate of 2.5 mm/min to observe the gap and 2 mm gap. Afterwards, the rate was adjusted to 25 mm/min to test the maximum tensile and Young's modulus. All data was recorded by a software program (Bluehill 2; Instron Corp).

### 2.6 Animals Tissue Collection and Dissociation for scRNA‐Seq

ScRNA‐seq experiment was performed by GENECHEM. Four types of FDL tendons (young male rat, aged male rat, young female rat, aged female rat; each group has 2 tendons) were surgically removed and kept in MACS Tissue Storage Solution (Miltenyi Biotec) until processing. The tissue samples were first washed with PBS, cut into small pieces (approximately 1 mm^3^) on ice and enzymatically digested with 1 mg/mL collagenase I (Gibco), 1 mg/mL collagenase II, 60 U/mL Hyaluronidase (Sigma), 10 U/mL Liberase (Roche) and 0.02 mg/mL DNase I (Roche) for 90 min at 37°C, with agitation. After digestion, samples were sieved through a 100 μm and 40 μm cell strainer, and centrifuged at 300 g for 5 min. Afterwards, red blood cell lysis buffer (Gibco) was used to lyse red blood cells. Finally, the cells were re‐suspended in PBS containing 0.04% BSA, stained and counted.

### 2.7 scRNA‐Seq Library Construction

Individually barcoded single‐cell RNA sequencing (scRNA‐Seq) libraries were prepared using the DNBelab C series high‐throughput Single‐cell RNA Library Prep kit (Version 2.0) in accordance with the protocol provided by the manufacturer (MGI). In brief, the processes of sample partitioning and molecular barcoding were conducted on the C4 instrument. For this step, cellular suspensions, P50 Oil, and Cell Beads‐V2 were loaded onto a C4 scRNA chip to form an emulsion. After that, reverse transcription was carried out on the RNA extracted from the barcoded cells, and sequencing libraries were constructed using reagents from the DNBelab C single‐cell RNA library kit V2.0 (MGI). Finally, sequencing was implemented with the DNBSEQ‐T7RS platform following the manufacturer's guidelines.

### 2.8 scRNA‐Seq Quality Control and Data Analysis

We used SCANPY to convert the single‐cell gene expression matrices from each dataset into annotated data objects and performed the following quality control steps: cells with fewer than 200 detected genes and cells with mitochondrial gene expression exceeding 25% were removed; the total gene expression counts for each cell were normalized to a fixed target value. Subsequently, we performed clustering analysis on the quality‐controlled cells. The FindAllMarkers method was applied to identify marker genes for each cluster, and these marker genes were compared with known cell‐type markers in the PanglaoDB database to annotate the cell types. Based on the annotation results, the cells were further categorized into major and subtypes, followed by functional and characteristic analyses at both levels. In addition, we conducted cell–cell communication and interaction analyses using CellChat and CellPhoneDB to uncover potential ligand–receptor signaling pathways and biological relationships among different cell types. To visualize the expression patterns of key genes across cell types, we generated gene expression heatmaps using SCANPY and Seaborn, which intuitively display the differential expression and distribution of representative marker genes across clusters.

### 2.9 Immunohistochemical Staining and Immunofluorescence

Tendon tissues were harvested and fixed in 4% paraformaldehyde for 24 h at room temperature. Then the samples were dehydrated and embedded into paraffin blocks. 5 μm sections were cut on a paraffin‐slicing machine and rehydrated. After blocking, the slides were incubated overnight at 4°C with a rabbit anti‐Creb3l1 antibody (1:200, Boster, Wuhan, China) a rabbit anti‐Sparc antibody (1:500, Abcam, Cambridge, UK) and a rabbit anti‐Collagen I antibody (1:500, Abcam) respectively for immunohistochemical staining. The slides for immunofluorescence were incubated with a rabbit anti‐CD86 antibody (1:500, Proteintech, Wuhan, China) and a mouse anti‐CD206 antibody (1:500, Proteintech) together. Then the slides were further incubated with secondary antibody at 37°C for 2 h. For immunohistochemical staining, 3,3‐ diaminobenzidine (DAB, Sigma, St. Louis, MO, USA) and hematoxylin were used. For immunofluorescence, DAPI was used to label cell nuclei. Finally, the slides were sealed and observed under a microscope (Leica DMR 3000; Leica, Bensheim, Germany).

### 2.10 H&E and Masson Staining

The sections of tendon tissues were rehydrated like mentioned above to conduct H&E and Masson staining. The staining steps were carried out according to the instructions of the reagent kits (Solarbio, Beijing, China). Finally, the slides were observed under a microscope (Leica).

### 2.11 Transmission Electron Microscope (TEM)

Fresh tendon tissues (1 mm^3^) from young male rats, aged male rats, young female rats, and aged female rats were fixed in 2.5% glutaraldehyde at 4°C for 12 h, rinsed with 0.1 M PBS, then post‐fixed in 1% osmium tetroxide for 1–2 h. After graded ethanol dehydration, tissues were infiltrated and embedded in epoxy resin, polymerized at 60°C for 24 h. Ultra‐thin sections (60–80 nm) were cut, collected on copper grids, stained with uranyl acetate and lead citrate. Sections were observed under a TEM at 80 kV, and representative images were captured.

### 2.12 Construction of Lentivirus

Creb3l1‐overexpressing lentiviruses (LV‐Creb3l1) and control lentiviruses (LV‐NC) were both purchased from Synbio Technologies (Suzhou, Jiangsu, China). Creb3l1 was obtained via PCR amplification of wild‐type cDNA (NM_001005562.2). The cDNA was subcloned into the pLVX‐AcGFP1‐N1 lentiviral vector using the *Eco*RI and *Bam*HI restriction enzyme, and the recombinant virus was packaged using lentivector expression systems. The pLVX‐AcGFP1‐N1 vehicle vector (LV‐NC) was used as an experimental control.

### 2.13 Culture of Primary Tenocytes

About 8‐week‐old rats were used and euthanized, and their tendon tissues were removed and washed with sterile PBS. Then the tendons were cut into about 2 mm × 2 mm × 2 mm pieces and placed on the culture dishes in a cell incubator (37°C, 5% CO_2_) with complete medium (90% DMEM, 10% FBS, 100 mg/mL streptomycin, 100 U/mL penicillin). About 7 days later, the tenocytes migrated from pieces and proliferated rapidly. Tissues were removed and the cells were passaged for the following experiments.

### 2.14 In Vitro Transfection Efficiency and Cell Viability

Tenocytes were seeded in a 24‐well plate at an initial density of 4 × 10^4^ cells/well. When the coverage ups to about 70%–80%, transient transfections were conducted by LV‐NC containing 4 × 10^5^ viral particles per well. Cells without transfection were used as control. After 48 h, the medium was replaced by fresh complete medium, and the cells were observed with fluorescence microscopy (Leica). Finally, the control cells and cells with transfection were tested on a flow cytometer (Attune NxT, Invitrogen). CCK‐8 (Servicebio, Wuhan, Hubei, China) was used to detect the viability of rat tenocytes when treated with LV‐NC. Cells were seeded in the 96‐well plate and then transfected with LV‐NC. After 24 h and 48 h, the specific steps for cell viability detection were carried out according to the experimental instructions.

### 2.15 Small Interfering RNA (siRNA) Transfection

Small interfering RNA against *Creb3l1* (si‐Creb3l1) and a non‐targeting control (si‐NC) were transfected into primary tenocytes at 60%–70% confluence with Lipofectamine RNAiMAX (Invitrogen) according to the manufacturer's instructions. Cells were collected 48 h later for qPCR and SA‐β‐gal staining.

### 2.16 Senescence‐Associated β‐Galactosidase (SA‐β‐Gal) Staining

Cellular senescence was assessed with a SA‐β‐gal staining kit (Beyotime, Shanghai, China) following the manufacturer's protocol. si‐NC and si‐Creb3l1 tenocytes were fixed and stained at 37°C overnight in a CO_2_‐free incubator, and the percentage of blue‐stained SA‐β‐gal‐positive cells was quantified from three random fields per group.

### 2.17 Protein Isolation and Western Blotting

Tenocytes transfected with LV‐NC and LV‐Creb3l1 were lysed in radioimmunoprecipitation assay (RIPA) lysis buffer (Beyotime). Protein concentrations were determined using an enhanced BCA protein assay kit (Beyotime). Proteins were separated via 12% sodium dodecyl sulfate‐polyacrylamide gel electrophoresis (SDS‐PAGE) and transferred to a polyvinylidene difluoride (PVDF) membrane (Millipore, Massachusetts, USA). The membranes were then blocked with blocking buffer (Beyotime) for 2 h at room temperature and incubated with a rabbit anti‐Sparc antibody (1:5000, Abcam), a rabbit anti‐Collagen I antibody (1:2000, Abcam), and a rabbit anti‐Creb3l1 antibody (1:2000, Boster) overnight at 4°C. After washing thrice (15 min each) with Tris‐buffered saline containing 0.1% Tween (TBST), blots were developed for visualization by using an Odyssey infrared imaging system (LI‐COR, Lincoln, NE, USA). Optical density of each band on the membrane was quantitatively determined with Image J.

### 2.18 Quantitative PCR

Total RNA was isolated from rat tenocytes transfected with LV‐NC and LV‐Creb3l1 using Trizol total RNA isolation reagent (Life Technologies). cDNA was synthesized using the HiScript III RT SuperMix for qPCR (+gDNA wiper) kit (Vazyme, Nanjing, Jiangsu, China). Real‐time quantitative polymerase chain reactions (qPCR) were used according to the instruction from ChamQ Universal SYBR qPCR Master Mix (Vazyme). We designed the primers depending on sequences from the Genebank database. The *Creb3l1* gene was amplified with 5′‐ACGCCGTCTTGGAACCTTT‐3′ and 5′‐TTGAGGAAATCCGACTCATTCA‐3′. The *Sparc* gene was amplified with 5′‐GTGATCTAAGTTCACGCCTCCTG‐3′ and 5′‐CTTGGGTTCTGGTTTCTTTATTTCT‐3′. The *Col1a1* gene was amplified with 5′‐AAGGCAATGCTGAATCGTCC‐3′ and 5′‐TGGTTAGGCTCCTTCAATAGTCC‐3′. The *beta‐actin* gene was amplified with 5′‐GACGTTGACATCCGTAAAGACC‐3′ and 5′‐GCTAGGAGCCAGGGCAGTA‐3′. The *p16* gene was amplified with 5′‐CGGGTCACCGACAGGCATA‐3′ and 5′‐TCTCGCGTTGCCAGAAGTG‐3′. The *p21* gene was amplified with 5′‐GCAAAGTATGCCGTCGTCTGT‐3′ and 5′‐GTCAAAGTTCCACCGTTCTCG‐3′. A melting curve was performed for each gene to ensure the specificity of the PCR. The target gene expression level relative to that of the β‐actin was expressed as 2^ΔCt^ = 2^Ctm‐Ctn^.

### 2.19 In Silico Prediction of Creb3l1 Binding to Col1a1 and Sparc Promoters

To explore whether Creb3l1 could directly bind the *Col1a1* and *Sparc* promoters, we adopted a previously reported strategy that combines promoter motif prediction with protein–DNA molecular docking (Antonieto et al. 2025). Potential Creb3l1 binding sites within the two promoters were identified with FIMO (MEME Suite) using the Creb3l1 motif MA0839.2 from JASPAR, yielding 13 candidate fragments (Grant et al. 2011; Rauluseviciute et al. 2024). The bZIP domain of rat Creb3l1 was modeled by AlphaFold and used as the docking receptor, and the candidate DNA fragments were built as B‐DNA duplexes with Web 3DNA (Jumper et al. 2021; Li et al. 2019). Molecular docking between the Creb3l1 bZIP model and each DNA fragment was performed with HDOCKlite v1.1, and the resulting protein–DNA interfaces were evaluated with Biotite (Kunzmann and Hamacher 2018; Yan et al. 2017). The FIMO, HDOCK, and Biotite results were then integrated to rank the candidate binding sites.

### 2.20 Dual‐Luciferase Reporter Assay

The wild‐type *Col1a1* and *Sparc* promoter regions containing the predicted Creb3l1 binding sites, as well as constructs in which these sites were mutated (Mut1–Mut4), were cloned into the pGL3‐basic vector upstream of the firefly luciferase gene. Tenocytes were co‐transfected with each reporter construct together with a *Creb3l1*‐overexpression vector (or the empty vector as control) and the pRL‐TK *Renilla* luciferase plasmid as an internal reference. After 48 h, firefly and *Renilla* luciferase activities were measured with a dual‐luciferase reporter assay system (Beyotime), and relative promoter activity was expressed as the firefly/*Renilla* ratio (*n* = 3–4).

### 2.21 Establishment of Rat Flexor Tendon Injury Model

A total of 18 aged male SD rats (36 tendons) and 18 aged female SD rats (36 tendons) were used in the rat FDL tendon injury model (No. S20250920‐006) and then equally divided into three groups: surgical repair (Control), surgical repair and injected with LV‐NC, surgical repair and injected with LV‐Creb3l1. Each tendon was injected at a dose of 2 × 10^9^ viral particles (Tang et al. 2008). All twelve samples in every group were used for the detection of gliding. Then nine samples were anatomically and biomechanically tested, and the other three samples were used for histological analysis without dissection.

For surgical procedure, animals were anesthetized with 3 mL/kg 2% pentobarbital sodium by intraperitoneal injection and 50% lidocaine hydrochloride solution containing 1% epinephrine was injected into the palmaris (0.05 mL/palm) to prevent serious bleeding. Flexor tendon was transected completely with a sharp surgical blade and was subjected to above treatments. After injection, each tendon was repaired with a 2‐strand Kessler's method and a circle repair by using 5–0 polypropylene sutures (Prolene, Ethicon, Somerville, NJ, USA). Finally, the skin was closed by 3–0 nylon running sutures (HOLYCON, Nantong, Jiangsu, China).

### 2.22 Gliding Function Testing

After 3 weeks, rat hind limbs were transected at the knee joint with euthanasia. The proximal flexor tendon along the tibia was isolated proximal to the tarsal tunnel, followed by tibial removal and paw dissection. The tendon's proximal end was secured with two injection needles (avoiding tendon penetration), and a single‐strand circular suture was used to facilitate tendon gliding and toe flexion. Specifically, one end of the suture was passed through the musculotendinous junction, while the other was threaded through a weight‐holding pouch. Tendon loading was applied via free‐hanging weights (0, 5, 10, 15, or 20 g). Starting with the toe in full extension, passive flexion was induced by proximal weight pull. The flexion angle—defined as the angle between the longest toe and horizontal plane—was recorded under each load using a digital camera mounted on a phone bracket positioned beside the paw.

### 2.23 Biomechanical Testing

Before dissection, the morphology and appearance of the adhesion were observed. Afterwards, repaired tendons were gently dissected from surrounding tissues and fully excised along their entire length for tensile strength testing. The tendon's two ends were secured in the lower and upper clamps respectively, with the repair site centered between the clamps on a testing machine (Model 3365; Instron Corp.). The upper clamp was then pulled upward at a constant speed of 25 mm/min until tendon rupture occurred. A load–displacement curve was recorded via Instron Series IX testing software, where a sharp decline indicated failure of the repaired site.

### 2.24 Statistical Analysis

Student *t*‐test was utilized to identify significant differences between the two groups, such as the tendon weight, the force of generating the gap, the force of 2 mm gap, the maximum tensile, Young's modulus, cell viability, expression of Creb3l1, Sparc, Colla1 and etc. For the data in three or more than three groups, such as gliding angel, ultimate strength and etc., one‐way analysis of variance was performed. Once the test was of statistical significance, the comparison between any two pair of groups was made with Tukey's multiple comparisons test. Statistical analyses were performed with use of GraphPad Prism (version 9.5.0), and the level of significance was set at *p* < 0.05.

## 3 Results

### 3.1 Temporal and Age‐Related Trends in Tendon Injuries due to Mechanical Force Exposure

**FIGURE 1:**
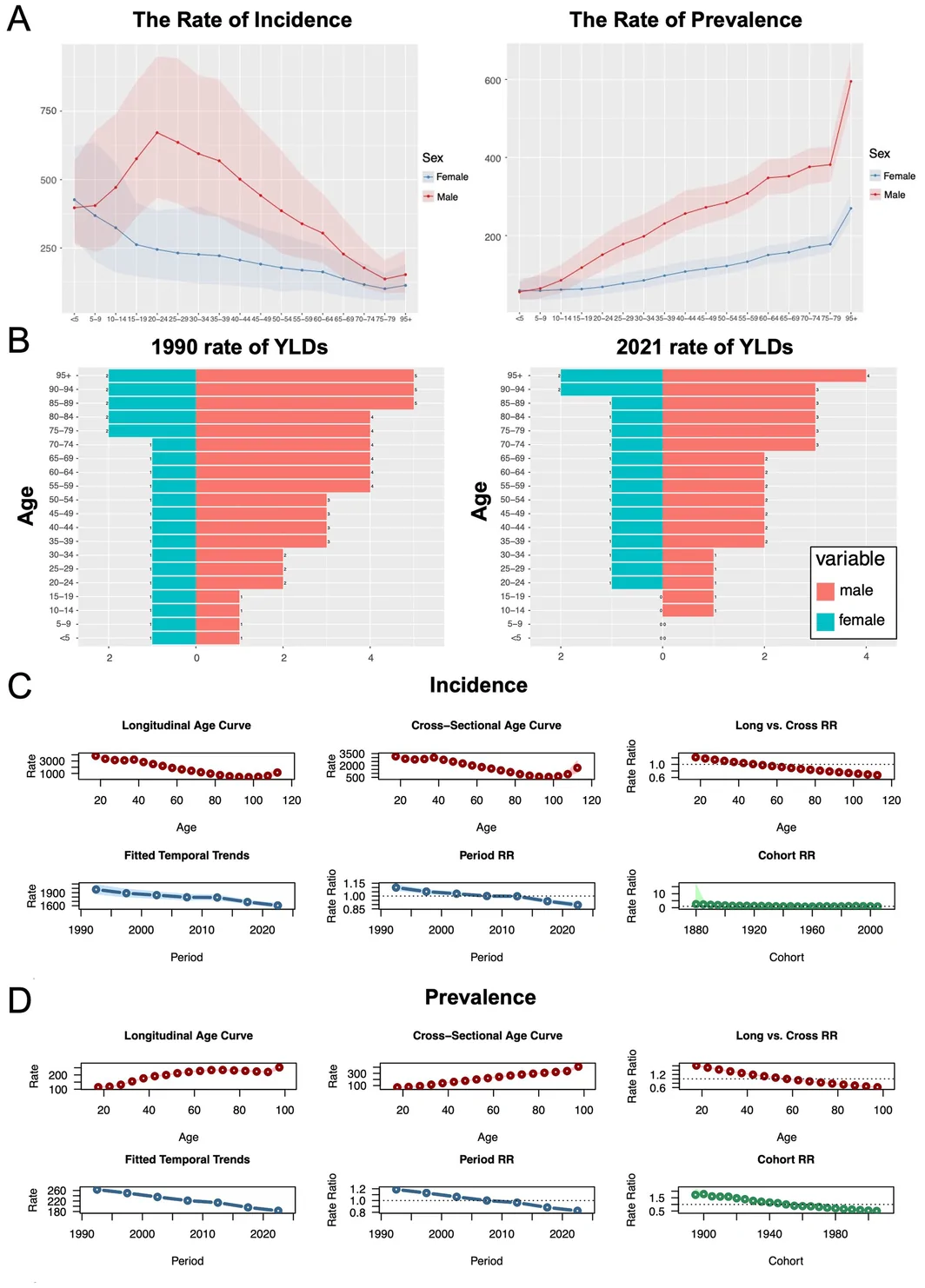
Impact of mechanical force on the disease burden of tendon injuries. (A) Incidence and prevalence of mechanically induced tendon injuries among males and females worldwide in 2021. (B) Years of healthy life lost to disability (YLD) from tendon injuries caused by mechanical force among males and females in 1990 and 2021. (C) Age‐Period‐Cohort model of incidence for tendon injuries attributable to mechanical force worldwide in 2021. (D) Age‐Period‐Cohort model of prevalence for tendon injuries attributable to mechanical force worldwide in 2021.

By 2021, the incidence of tendon injuries caused by mechanical force exhibited an overall downward trend with increasing age, which was closely linked to social progress and the widespread adoption of risk education. In contrast, both the prevalence and years of life lost due to disability (YLD) rose significantly with age. Compared with 1990, the overall YLD showed an aging trend, indicating a close relationship between mechanical force–induced tendon injuries and older age. Nevertheless, with the further promotion of risk education and advances in industrial automation, the incidence rate was expected to decrease substantially (Figure 1A,B). To analyze the changes in incidence and prevalence across age, period, and birth cohort, we employed an APC model. The APC model showed that in the incidence of mechanical force–induced tendon injuries, both the age effect and period effect decreased with advancing age, while birth cohort changes were not pronounced (Figure 1C). By contrast, in prevalence, the age effect increased with age, but the period effect and birth cohort both declined (Figure 1D). Economic growth and improvements in medical technology significantly reduced the incidence of these injuries without affecting prevalence risk. Finally, the mixed‐effects APC model effectively captured the trends of all three effects in prevalence (Yue et al. 2025).

### 3.2 Spatial Distribution of the Disease Burden of Tendon Injuries Caused by Mechanical Force Exposure

From 1990 to 2021, the age‐standardized incidence rate (ASIR) and age‐standardized prevalence rate (ASPR) of tendon injuries caused by mechanical force exposure in male patients both exhibited a gradual downward trend (Figure S1A,B). In the 21 global regions, the disease burden metrics showed an inverted “V” relationship with the SDI. When the SDI was below 0.75, ASIR and ASPR gradually increased; once it exceeded 0.75, they began to decline (Figure S1C,D). As a country's SDI increased, the disease burden metrics decreased correspondingly (Sang et al. 2022) (Figure S1E,F).

### 3.3 Age‐Related Differences in Mechanical Property

**FIGURE 2:**
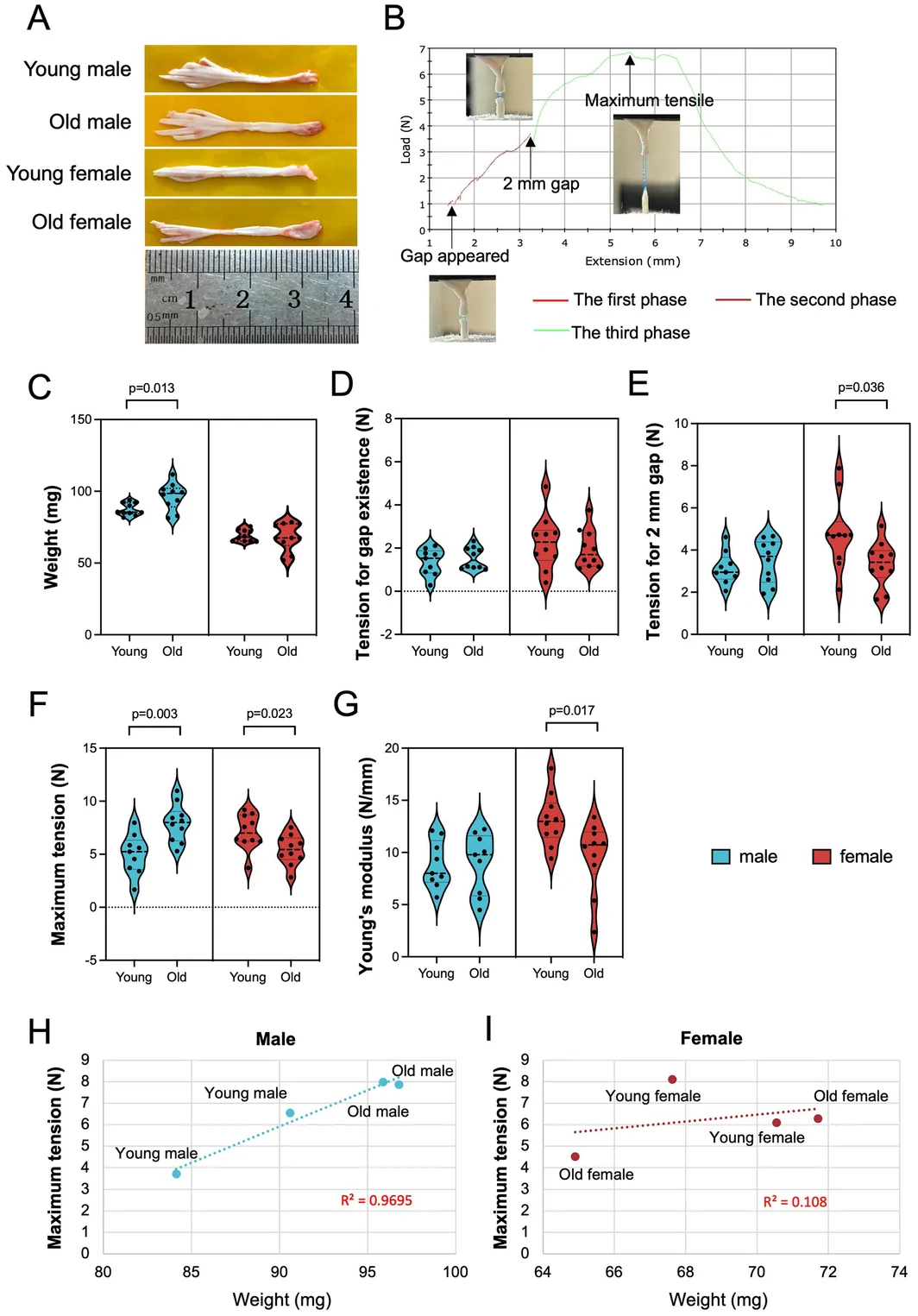
Age‐related differences in tendon mechanical property. (A) The length of FDL tendons from the muscle‐tendon junction to the site of proximal phalanx in young male, aged male, young female, and aged female groups. (B) The curve and condition of repaired tendon during the detection for the force to generate the gap, 2 mm gap, and the maximum tensile. (C) The weight, (D) tension for gap existence, (E) tension for 2 mm gap, (F) maximum tension and (G) Young's modulus of rat FDL tendons in young male, aged male, young female, and aged female groups. The correlation between the maximum tension and tendon weight in (H) male and (I) female rats.

The average length of FDL tendons was about 3.5 cm and there was no obvious difference among young male, aged male, young female, and aged female groups (Figure 2A). The graphs of three phases for gap testing showed in Figure 2B.

#### 3.3.1 For Male Group

The weight of tendons in aged male group significantly increased to 96.3 ± 9.4 mg as the weight of tendon in young male group was about 87.4 ± 4.3 mg (*p* = 0.013). One sample in young male group ruptured after the 100 stretching cycles. Therefore, there were 9 samples in young male group evaluated in the following tests. The force of generating the gap, the force of 2 mm gap, and Young's modulus was similar between two groups. Only the maximum tensile strength of tendons in aged male group increased when compared to that of young male group (young male: 5.0 ± 1.9 N; aged male: 7.9 ± 1.8 N; *p* = 0.003) (Figure 2C–G). By analyzing the correlation between tendon weight and maximum tensile strength, we found that the increase in tension in aged male rats was closely related to the increase in tendon weight (*R* = 0.9695, Figure 2H).

#### 3.3.2 For Female Group

The average weight of tendons in the female group changed from 69.1 ± 4.0 mg to 68.3 ± 9.4 mg with aging. The force of 2 mm gap (3.3 ± 1.1 N, *p* = 0.036), maximum tensile (5.4 ± 1.4 N, *p* = 0.023) and Young's modulus (9.6 ± 3.4 N/mm, *p* = 0.017) all dramatically decreased in the aged female group, as those in the young female group were 4.8 ± 1.7 N, 7.1 ± 1.7 N and 13.1 ± 2.5 N/mm respectively. The tension for generating the gap was no difference between the two groups (Figure 2C–G). Although there was a slight decrease in the weight of tendons in aged female rats, there was no close correlation with a significant reduction in maximum tensile (*R* = 0.108, Figure 2I).

### 3.4 Age‐Related Differences of Rat Tendons in scRNA‐Seq

To evaluate the age‐related changes in tendons, we collected tendons from male and female rats at 8 weeks‐ and 18 months‐old and performed scRNA seq. After quality control analysis, filtration, and double removal, tendon tissues in young male rats had 3802 cells, tendon tissues in aged male rats had 3833 cells, tendon tissues in young female rats had 4496 cells, and tendon tissues in aged female rats had 7521 cells for characterization analysis.

To determine the identity of cell types, we used cell type specific markers previously reported in the literature (de Micheli et al. 2020; Kendal et al. 2020). Overall, the most common cell types were fibroblasts (Myoc, Dcn, Comp), stromal cells (Tagln, Myl9), epithelial cells (CD74, Plvap, Fabp4, Flt1), and immune cells (C1qa, Lyz2, C1qb) (Figure S2A,C). Fibroblasts were divided into seven subgroups of fibroblasts and three subgroups of chondrocytes. Epithelial cells were mainly divided into vascular smooth muscle cells, vascular endothelial cells, and endothelial cells. Macrophages accounted for the highest proportion in immune cells, in addition to DC cells, mast cells, and T cells (Figure S2B,D).

To improve the biological resolution of the fibroblast/stromal compartment, we further re‐clustered and re‐annotated these cells using tendon‐relevant marker genes. This refined annotation resolved multiple tendon stromal populations, including tenocyte‐like cells, epitenon‐like cells, ECM fibroblasts, repair fibroblasts, progenitor‐like fibroblasts, stress fibroblasts, and specialized fibroblasts (Figure S3A,B). Marker validation showed that tenocyte‐like cells were enriched for Scx, Tnmd, Kera, Col1a1, and Comp, whereas epitenon‐like cells showed higher expression of Col14a1, Pi16, Dpt, and C7 (Figure S3C).

**FIGURE 3:**
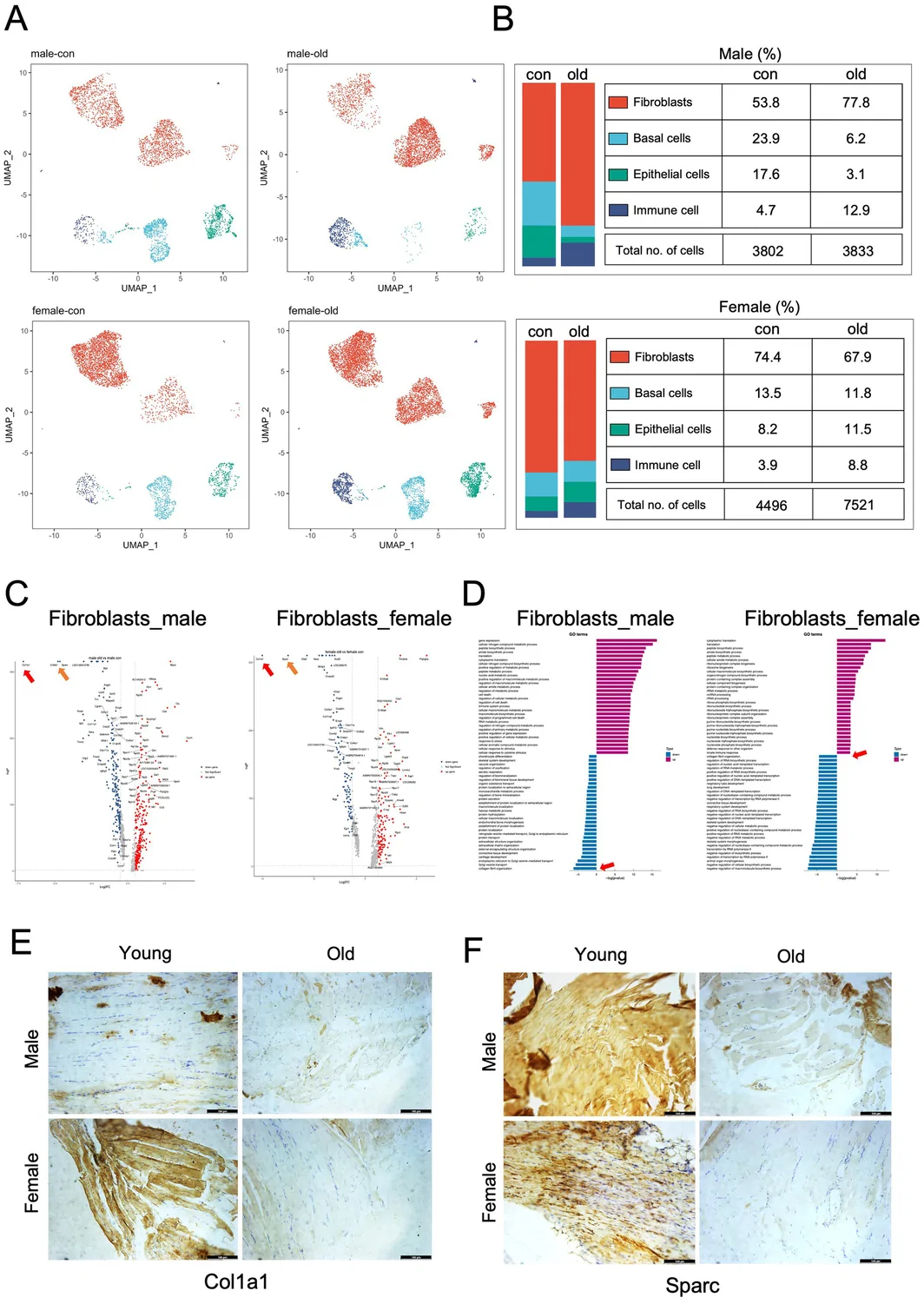
Age‐related changes in tendon cell populations. (A) UMAP plot and (B) percentages of tendon cells with different genders by age. (C) Volcano diagram of differentially expressed genes of fibroblasts with different genders by age. (D) GO analysis of fibroblasts with different genders by age. The expression of (E) Col1a1 and (F) Sparc of tendon tissues with different genders by age through immunohistochemical staining.

In our analysis, increasing age did not significantly alter the cell count in the tendons of male rats, but significantly increased the cell count in female rats, reaching approximately 167%. For male rats, with increasing age, the cell numbers of fibroblasts and immune cells increased, with the proportion of fibroblasts rising from 53.8% to 77.8% and the proportion of immune cells rising from 4.7% to 12.9%. The decrease in cell numbers was observed in stromal cells and epithelial cells, with stromal cell numbers decreasing from 23.9% to 6.2% and epithelial cell numbers decreasing from 17.6% to 3.1%. For female rats, as they age, the number of cells increased in epithelial cells and immune cells, with the proportion of epithelial cells increasing from 8.2% to 11.5% and the proportion of immune cells increasing from 3.9% to 8.8%. The decrease in cell numbers was observed in fibroblasts and stromal cells, with the proportion of fibroblasts decreasing from 74.4% to 67.9% and the proportion of stromal cells decreasing from 13.5% to 11.8% (Figure 3A,B).

### 3.5 Differential Gene Analysis of Different Cells

We conducted differential gene analysis on cell types with a high proportion of quantity, including fibroblasts, macrophages, DC cells, endothelial cells, and vascular smooth muscle cells. We have discovered an interesting phenomenon where two genes, *Col1a1* and *Sparc*, were significantly downregulated in the aged tendon tissues of both male and female rats in these cells with a high proportion of numbers (Figure 3C and Figure S2E,F). GO analysis of fibroblasts revealed a significant reduction in collagen fiber synthesis in aged tendon tissues (Figure 3D). We performed immunohistochemical staining on the tendon tissues of male and female rats and found that, consistent with sequencing results, the Col1a1 and Sparc proteins in the tendon tissues of aged rats showed weak positive signals compared to those of young rats (Figure 3E,F).

Given the central role of collagen and extracellular matrix remodeling in tendon aging, we further examined the distribution of Creb3l1, Col1a1, and Sparc within the refined fibroblast/stromal populations. These genes were enriched within the tenocyte‐like/ECM‐rich fibroblast region (Figure S3D). In tenocyte‐like cells, Creb3l1, Col1a1, and Sparc expression decreased in aged rats compared with young controls in both female and male groups (Figure S3E), suggesting that aging impairs the ECM biosynthetic program of tenocyte‐like cells.

### 3.6 CellChat and Collagen Fiber Analysis

To evaluate the intercellular signaling network, we conducted CellChat analysis. The most significant changes in cellular signaling occurred in fibroblasts and immune cells in both male and female rats. From 8 weeks to 18 months, the weight of the interaction between fibroblasts and immune cells increased overall, but the weight of the interaction between fibroblasts and other cells such as endothelial cells, pericytes, and vascular smooth muscle cells significantly decreased or even disappeared (Figure S4A,B).

We observed the characteristics of collagen fibers and cells in tendons through H&E staining. It could be observed that the eosin staining in the tendons of aged male and female rats was lighter, indicating a decrease in alkaline components such as proteins, collagen fibers, extracellular matrix, etc. in the cytoplasm of aged tissues. The cells distributed in collagen fibers exhibited sparse, disordered, and shorter length characteristics in aged tissues when compared to the dense, elongated, and neatly arranged features in young tissues (Figure S4C,D). We used transmission electron microscopy to observe tendon tissues of both young and aged individuals and found that the arrangement of collagen fibers in young tendon tissues was denser in both males and females. There were varying degrees of gaps between collagen fibers in the aged tendon tissues (indicated by yellow arrows) (Figure S4E,F).

### 3.7 Transcription Factor Creb3l1 Regulated Col1a1 and Sparc in Fibroblasts

**FIGURE 4:**
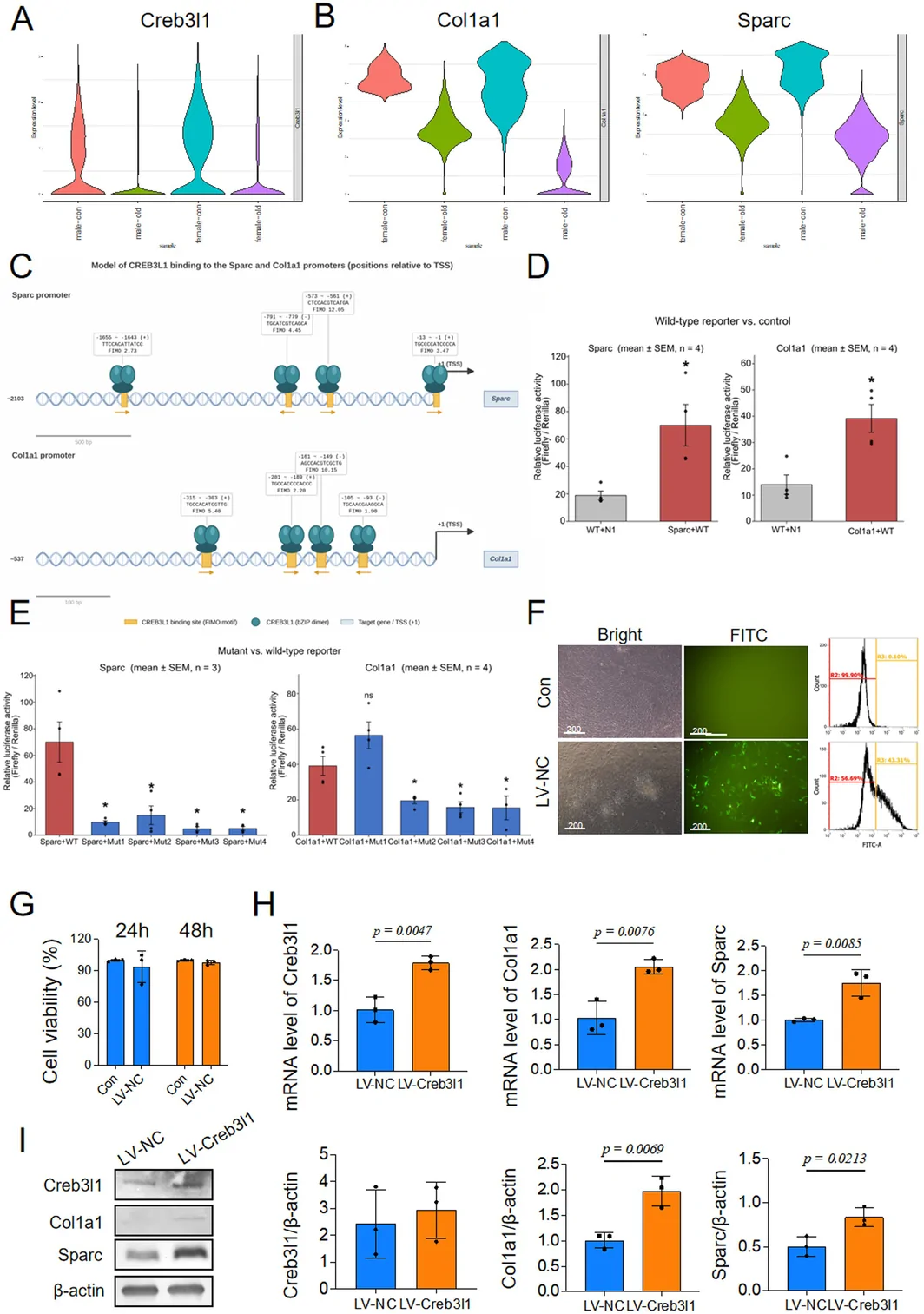
The regulatory effect of transcription factor Creb3l1 on Col1a1 and Sparc. In single‐cell sequencing data, the expression levels of (A) Creb3l1, (B) Col1a1 and Sparc in fibroblasts of rats of different genders at different ages. (C) Predicted Creb3l1 binding sites in the Sparc and Col1a1 promoters (positions relative to the TSS, +1), each labeled with sequence and FIMO score. Orange, Creb3l1 binding site; teal, Creb3l1 bZIP dimer. Scale bars, 500 bp (Sparc) and 100 bp (Col1a1). (D) Dual‐luciferase activity of wild‐type Sparc and Col1a1 promoter reporters with Creb3l1 versus control. (E) Wild‐type versus binding‐site mutant (Mut1–Mut4) reporters with Creb3l1. (F) Transfection efficiency of rat tenocytes transfected with LV‐NC for 48 h. (G) Cell viability of rat tenocytes transfected with LV‐NC for 24 h and 48 h. The (H) mRNA level and (I) protein of Creb3l1, Col1a1 and Sparc of rat tenocytes when transfected with LV‐NC and LV‐Creb3l1 after 48 h.

We performed differential gene analysis of common transcription factors in fibroblasts, with endothelial cells used for supplementary validation (Table 1) and found that the transcription factor Creb3l1 was downregulated in both genders of aged tendon tissues and in both types of cells (Figure 4A). The violin plots showed the decreased expression of *Col1a1* and *Sparc* in fibroblasts in aged tendon tissues (Figure 4B). Jaspar database was used to predict potential Creb3l1‐binding sites in the *Col1a1* and *Sparc* promoters (Figure 4C,D). To test whether Creb3l1 directly regulates these genes, Creb3l1 binding sites in the Col1a1 and Sparc promoters were predicted by FIMO and confirmed by molecular docking of the Creb3l1 bZIP domain (Figure 4C). In dual‐luciferase reporter assays, Creb3l1 significantly increased both Sparc and Col1a1 wild‐type promoter activity (Figure 4D, *n* = 4). Mutating the predicted sites abolished this activation for all four Sparc mutants and for Col1a1 mutants 2–4, whereas Col1a1 mutant 1 was unchanged (Figure 4E). These results indicate that Creb3l1 directly activates the Col1a1 and Sparc promoters. Rat tenocytes transfected with LV‐NC showed green fluorescence in the FITC channel and the transfection efficiency was about 43% (Figure 4F). Compared with non‐transfected cells, the cell viability of the transfected group did not significantly decrease at both 24 and 48 h after transfection (Figure 4G). By transfecting lentivirus overexpressing Creb3l1, we found that Col1a1 and Sparc significantly increased at both mRNA and protein levels. However, the increase in Creb3l1 itself was not significant at the protein level, but significant at the gene level (Figure 4H,I).

**TABLE 1:** Expression of transcription factors.

1	Crem	female‐con	0.177478290742384
2	Crem	female‐old	0.134518333315754
3	Crem	male‐con	0.257026220565932
4	Crem	male‐old	0.243286325826266
5	Creb3l1	female‐con	0.958250728536525
6	Creb3l1	female‐old	0.320529467117133
7	Creb3l1	male‐con	0.657037626509754
8	Creb3l1	male‐old	0.0916716976985902
9	Atf4	female‐con	1.54715546943621
10	Atf4	female‐old	1.28835009136522
11	Atf4	male‐con	1.62520812885052
12	Atf4	male‐old	1.9114560769125
13	Foxo1	female‐con	0.251630483798831
14	Foxo1	female‐old	0.192076676935371
15	Foxo1	male‐con	0.110512201290781
16	Foxo1	male‐old	0.147242277198841
17	Foxo3	female‐con	0.130669240793763
18	Foxo3	female‐old	0.0667689522594851
19	Foxo3	male‐con	0.0431059155959004
20	Foxo3	male‐old	0.0504480770821075
21	Nfkb1	female‐con	0.28249283051505
22	Nfkb1	female‐old	0.201034565623193
23	Nfkb1	male‐con	0.281424819187432
24	Nfkb1	male‐old	0.438868105273359
25	Hif1a	female‐con	0.669162926077349
26	Hif1a	female‐old	0.359316292938025
27	Hif1a	male‐con	0.667159780918543
28	Hif1a	male‐old	0.228440000231922
29	Runx1	female‐con	0.525502977202992
30	Runx1	female‐old	0.32408479001764
31	Runx1	male‐con	0.117781129326036
32	Runx1	male‐old	0.298465572272576
33	Runx2	female‐con	0.00943619643295823
34	Runx2	female‐old	0.0089016147590641
35	Runx2	male‐con	0.0160005762430395
36	Runx2	male‐old	0.0221811020250262
37	Runx3	female‐con	0.00129436612880104
38	Runx3	female‐old	0.000646426472548125
39	Runx3	male‐con	0.000785939930825795
40	Runx3	male‐old	0
41	Klf4	female‐con	1.59656020491961
42	Klf4	female‐old	1.53804071493916
43	Klf4	male‐con	2.01755102407327
44	Klf4	male‐old	2.74077875846207
45	Stat1	female‐con	0.0878708272903452
46	Stat1	female‐old	0.106986107333382
47	Stat1	male‐con	0.24304607886628
48	Stat1	male‐old	0.171586279647981
49	Stat3	female‐con	0.61056286951624
50	Stat3	female‐old	0.644716011341851
51	Stat3	male‐con	0.798151035908171
52	Stat3	male‐old	1.09995820577059
53	Stat6	female‐con	0.302829335974928
54	Stat6	female‐old	0.278992248527051
55	Stat6	male‐con	0.31055318832578
56	Stat6	male‐old	0.272181763119005
57	Fos	female‐con	2.5513384461292
58	Fos	female‐old	1.83667204768346
59	Fos	male‐con	2.3736403830989
60	Fos	male‐old	2.2716267966199
61	Jun	female‐con	1.80958903658353
62	Jun	female‐old	1.26523509376317
63	Jun	male‐con	2.33947627300095
64	Jun	male‐old	2.41720954321494

### 3.8 Overexpression of Creb3l1 Promoted the Repair of Injured Tendons in Aged Rats

#### 3.8.1 For Male Group

**FIGURE 5:**
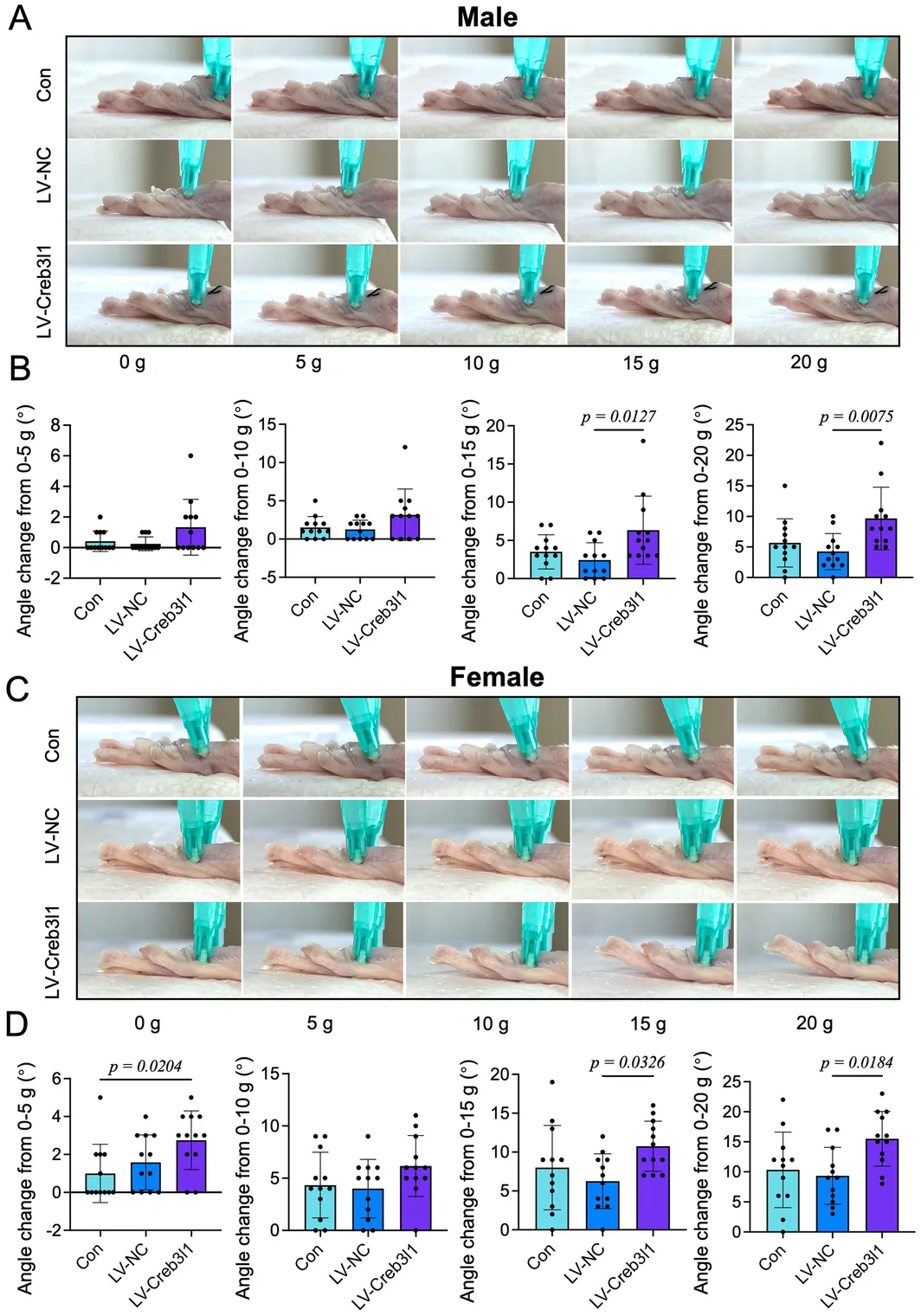
Overexpression of Creb3l1 promotes the gliding function of injured tendons in aged rats. Typical images and statistical difference of flexion angle change of rat FDL tendons in Con, LV‐NC and LV‐Creb3l1 groups with the weight enhanced for male (A, B) and female (C, D) rats. *n* = 12 in each group.

**FIGURE 6:**
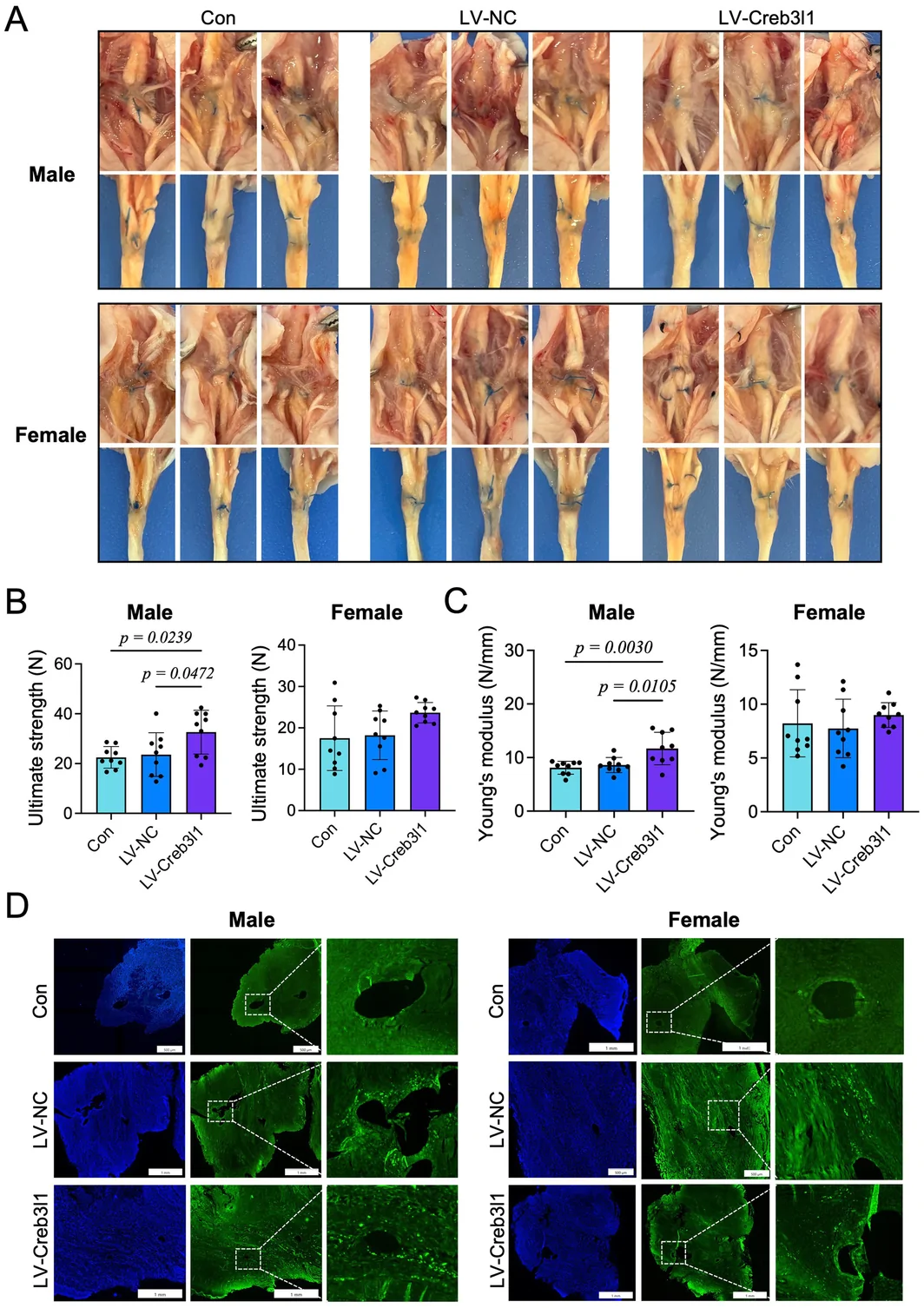
Overexpression of Creb3l1 promotes the healing of injured tendons in aged rats. (A) Pictures of healed rat FDL tendons after dissection in Con, LV‐NC and LV‐Creb3l1 groups for male and female rats. The ultimate strength (B) and Young's modulus (C) of rat FDL tendons in Con, LV‐NC and LV‐Creb3l1 groups for male and female rats (*n* = 9 in each group). (D) Representative EGFP expression images in the repaired tendon tissues in Con, LV‐NC and LV‐Creb3l1 groups for male and female rats.

At weeks 3, tissues containing tendon and adhesion were harvested. Gliding test was first conducted. With the weights increased, tendons in the LV‐Creb3l1 group showed the greatest flexion function among the three groups, especially at 15 and 20 g weights (Figure 5A,B). After tendon dissection, the healing status of the repaired tendons was first evaluated by gross observation. It was observed that the tendon stumps in the LV‐Creb3l1 group were almost connected by healing milky white tendinous tissue, whereas the tendon stumps in the Con and LV‐NC groups were mostly connected by translucent granulation tissue (Figure 6A). The healing strength of the tendons in the Con and LV‐NC groups reached 22.5 ± 4.3 N and 23.6 ± 8.8 N, respectively, while that of the LV‐Creb3l1 group was 32.6 ± 8.8 N, representing a significant increase of 145% and 138% with statistical differences (Figure 6B). For the elastic modulus, the value in the LV‐Creb3l1 group was 11.7 ± 3.0 N/mm, which was also significantly higher than those in the Con group (8.1 ± 1.2 N/mm) and the LV‐NC group (8.6 ± 1.4 N/mm) (Figure 6B,C).

#### 3.8.2 For Female Group

The mean flexion angles of tendons in the LV‐Creb3l1 group were higher than those in the two control groups under all weights conditions, with statistically significant differences observed at weights of 5, 15 and 20 g (Figure 5C,D). Like male rats, the tendon stump connections in the LV‐Creb3l1 group of female rats were more intact (Figure 6A). However, although the mean values of tensile force and elastic modulus of tendons in the LV‐Creb3l1 group were both higher than those in the other two control groups, no statistically significant differences were detected for these two indices (Figure 6B,C).

### 3.9 Overexpression of Creb3l1 Regulated Col1a1 and Sparc In Vivo

The transfection efficiency of lentivirus in tissues was evaluated by observing the GFP green fluorescence signals. As shown in Figure 6D, no specific green fluorescence was detected around the suture holes in the tendons of both male and female rats in the Con group, whereas a large number of green fluorescence cells were distributed around the suture holes in the tendons of rats in the LV‐NC and LV‐Creb3l1 groups. Immunohistochemical staining analysis revealed that the positive signals of Creb3l1, Col1a1, and Sparc proteins in the tendons of rats in the LV‐Creb3l1 group were all stronger than those in the other two control groups (Figure S5A).

### 3.10 The Changes of Histology and Macrophage Polarization in Repaired Tendons and the Effect of si‐Creb3l1 on Tenocytes

Compared with the tendons in the Con and LV‐NC groups, H&E and Masson staining revealed deeper eosin staining and collagen fiber staining in the tendons of the LV‐Creb3l1 group, with the fibers arranged in a dense, slender, and regular pattern (Figure S5B). To further define the role of Creb3l1 in tenocytes, *Creb3l1* was silenced with siRNA. si‐Creb3l1 markedly reduced *Creb3l1* mRNA compared with si‐NC (Figure S6A, *p* < 0.0001), accompanied by significantly decreased *Col1a1* and *Sparc* expression (Figure S6B,C). The senescence markers p16 and p21 showed an upward trend after knockdown, although the differences were not significant (Figure S6D,E). Consistently, SA‐β‐gal staining showed that Creb3l1 knockdown markedly increased the number of senescent, SA‐β‐gal‐positive (blue‐stained) tenocytes compared with the si‐NC group (Figure S6F). Quantification further confirmed that the proportion of SA‐β‐gal‐positive cells rose significantly from 9.4% in the si‐NC group to 23.7% in the si‐Creb3l1 group (Figure S6G, *p* = 0.0003). These results indicate that loss of *Creb3l1* downregulates *Col1a1* and *Sparc* and promotes tenocyte senescence (Figure S6). Our results revealed that CD86‐ and CD206‐positive cells were distributed in tendon and peritendinous tissues at 3 weeks post‐surgery in both male and female rats. Compared with the Con and LV‐NC groups, the number of CD86‐positive cells showed no obvious reduction in the LV‐Creb3l1 group, whereas the number of CD206‐positive cells was increased (Figure S7).

## 4 Discussion

In this study, we aimed to investigate the age‐related differences in mechanical properties of injured tendons, from the epidemiological trend to the animal model. Overall, as age increased, some biomechanical properties of tendons gradually weakened. Through single‐cell sequencing, we found that the expression levels of *Col1a1* and *Sparc* genes closely related to collagen production were significantly reduced in aged tendon tissue and showed sparse collagen fiber morphology. In addition, we found that the decrease in transcription factor Creb3l1 may be the reason for the decreased expression of *Col1a1* and *Sparc*. Overexpression of Creb3l1 can improve the repair effect of injured tendons in aged rats.

Based on the data from 21 regions and 204 countries worldwide, the influence on tendon injury from mechanical force increased with the age increment, involving the ASPR and YLDs. The ASPR and YLDs of the male group were both higher than that of the female group, which was related to the division of labor in families and the differences in the types of work for different genders. The ASPR increased sharply after the age of 75, which indicated that the risk exposure was higher with the decline of reaction ability with aging. Interestingly, the ASIR of both female and male groups typically decreased with age, with only the male group reaching its peak at the age of 20–24. The decline of ASIR might be related to the increase of SDI (Lippi et al. 2020; Li et al. 2020). With the increase of SDI and the development of medical resources, people's health conditions are improved, and the disease burden is reduced, thus affecting the change of incidence rate.

Compared with previous mouse tendon aging studies that mainly focused on mechanical alterations, fatigue loading, ex vivo mechanical responses, and general gender‐related phenotypic differences, we performed mechanical tests on ex vivo tendon tissues using rat models to further supplement the research background regarding mechanical property changes of rat tendons during aging. This can validate the results from single‐cell RNA sequencing and provide a research basis for the in vivo regulation of Creb3l1. In addition, the rat tendon model possesses several other advantages. First, the flexor tendons of rats are larger in volume and mass and can withstand greater mechanical loading. For instance, during the 100 stretching cycles test in this study, rat tendons maintained a relatively stable morphological state, allowing subtle differences in tendon gap formation after rupture to be clearly observed among rats of different ages. Second, the larger size of rat tendons permits needle insertion into the tendon tissue by using a microinjector in animal experiments, effectively avoiding drug leakage and loss. This provides reliable experimental data for exploring the regulatory mechanism of Creb3l1 on the expression of Col1a1 and Sparc. Third, previous mouse studies have merely identified the existence of sex differences in tendon aging, yet failed to quantify the divergent mechanical properties, such as tendon weight and maximum tensile force between males and females. Finally, this study also provides references for other researchers to understand the differences in aging‐induced changes in tendon mechanical properties between mice and rats.

To gain a clearer understanding of age‐related differences in mechanical property of injured tendons, we conducted a series of biomechanical tests on the injured tendons of aged and young rats, including males and females. Application of 100 cycles from 1 to 10 N was to simulate the situation where tendons in vivo were repeatedly stimulated by mechanical forces, which was consistent with the selection of “mechanical force” as an exposure factor in epidemiological analysis. This stretching cycle method is commonly used to detect the response of tendons or tenocytes to mechanical forces (Ishizaki et al. 2024; Burk et al. 2016; Wang et al. 2003; Lavagnino et al. 2014). Usually, gap formation between broken ends of tendons was measured to evaluate the efficacy and stabilities of repair sutures before (Yang and Zhou 2019; Wu and Tang 2012). In this study, we measured the gap and maximum tensile to evaluate the ability of rat tendons with different ages and genders to resist suture tension after force stimulation. There was a significant difference in tendon weight between males and females, and the weight of tendons in male rats further increased with age, while the weight of tendons in females remained stable. In the following biomechanical tests, we found significant differences between males and females. The tendons of female rats showed a significant decrease in their ability to resist gaps, tensile strength, and elastic modulus with increasing age. Michael Lavagnino and his colleagues also found the contraction function significantly decreased with age increasing in SD rat model (Lavagnino et al. 2014). However, in male rats, only the tensile strength significantly increased, while there was no significant change in the gap resistance and elastic modulus. It was difficult to say that the biomechanical properties of tendons in aged male rats were superior to those in young male rats, as the increase in tensile strength may be closely related to the increase in tendon weight (Kayser et al. 2022). We speculate that the male rats might have a greater ability to resist the degeneration of tendon tissue function caused by aging than female rats.

While biomechanical analyses failed to detect significant degenerative changes in the tendon tissues of male rats, single‐cell sequencing revealed age‐related tendon degeneration in both male and female rats, with the expression profiles of associated genes being highly consistent between the two genders. In tendon tissues of aged individuals, the expression levels of *Col1a1* and *Sparc* were markedly downregulated in most cell types; this reduction was particularly pronounced in fibroblasts, which constitute the most abundant cell population in tendons and exhibit a concomitant decrease in collagen fiber synthesis. Col1a1 encodes the α1 chain of Type I collagen. Its expression product assembles with the α2 chain encoded by Col1a2 to form a Type I collagen heterotrimer, which undergoes propeptide cleavage and then self‐assembles into well‐organized collagen fibers, serving as the structural and mechanical foundation of tendons. Fibroblasts exhibit high‐level expression of Col1a1. The downregulation of its transcription directly reduces collagen synthesis and disrupts fiber alignment, representing a key molecular hallmark of age‐related tendon degeneration (Gelse et al. 2003; Prockop and Kivirikko 1995; Wang et al. 2021). As a collagen‐binding matricellular protein, Sparc maintains tendon homeostasis by regulating the assembly of Type I collagen fibers and promoting the mechanotransduction response of fibroblasts. Deletion or mutation of Sparc leads to impaired tendon development and maturation (Gehwolf et al. 2016; Wang et al. 2021). Single‐cell sequencing also revealed that the transcription factor Creb3l1 is downregulated in aged tendon tissues, and our validation experiments found this factor can regulate the transcription of Col1a1 and Sparc via direct binding to their respective promoter regions. The accumulation of oxidative stress and inflammatory factors associated with the aging process significantly represses the expression of Creb3l1 (Jeong et al. 2025). In addition, certain growth factors, such as TGF‐β1 and BMP‐2, can directly modulate the transcriptional activity of its gene (Chen et al. 2014; Murakami et al. 2009). We speculate that the decrease in Creb3l1 expression is closely related to tissue aging.

Recent mouse tendon single‐cell studies further support the need to distinguish tenocytes and epitenon cells as biologically distinct tendon stromal populations. Korcari et al. (2023) reported age‐associated remodeling of mouse tendon, including impairment of ECM biosynthetic tenocyte programs, whereas Nichols et al. (2025) demonstrated that epitenon‐derived progenitors make distinct spatial and functional contributions to flexor tendon healing and fibrosis. To place our rat data in this context, we added a literature‐guided comparison between published mouse tendon studies and the present rat single‐cell dataset (Table S1). This comparison shows that tenocyte/tenocyte‐like and epitenon/epitenon‐like populations can be identified across rodent tendon studies using overlapping stromal markers. Together, these findings suggest that aging‐associated impairment of matrix‐producing tendon stromal cells is at least partially conserved across mouse and rat, while our study further links this process to the Creb3l1‐Col1a1/Sparc axis in aged rat tenocyte‐like cells.

Based on the above results, we overexpressed Creb3l1 to evaluate the repair effect of injured tendons in aged rats. We found that the gliding function of tendons was improved in both male and female rats; however, the healing strength was significantly enhanced only in male rats. Although the mean value of tendon healing strength in female rats showed an increasing trend, the difference was not statistically significant. We speculate that age‐related degeneration of tendons is more pronounced in female rats, or it is associated with more factors such as estrogen levels. Newly synthesized Col1 primarily exists as soluble, uncrosslinked precursors. The formation of stable covalent crosslinks relies on crosslinking enzymes including LOX, BMP‐1, and PLOD. Previous studies have demonstrated that estrogen modulates LOX expression, while estrogen deprivation might inhibit LOX activity (Balgobin et al. 2013; Lee et al. 2015; Li et al. 2017). We therefore hypothesized that although Creb3l1 overexpression upregulated Col1a1 expression in female rats, the deficiency of functionally active crosslinking enzymes prevented the assembly of stable collagen fibers that can be effectively integrated into the ECM network. In addition, after treatment with LV‐Creb3l1, the proportion of M2 macrophages in female rats increased by less than 150%, while that in male rats rose to approximately 230%. We also speculated that the unsatisfactory healing effect in female rats was also closely associated with the immune microenvironment. However, further investigations are required to verify this hypothesis.

This study has several limitations. First, due to tendon injury being a minor disease, some countries and regions in the GBD database do not have complete data about tendon injury. Second, we selected the “mechanical force” as the exposure factor for tendon injury, but there are many other exposure factors that might cause tendon injury, such as obesity, chronic inflammatory conditions, fluoroquinolone usage, etc. (Arnold et al. 2022). Third, tendon injury includes acute injury and chronic injury, like tendinopathy, tendon tear injury, or acute tendon rupture. It's difficult for us to define specific tendons and specific diseases during epidemiological investigations. Fourth, the ex vivo tendon repair model cannot fully reflect age‐related mechanical degeneration of tendons, as it is closely associated with tendon suture. We performed such experiments to exclude the interference of in vivo inflammatory infiltration, vascular regulation and systemic metabolic factors, and to compare the differences in biomechanical properties between young and aged tendon tissues under standardized suture repair conditions as far as possible. Fifth, when we conducted tests on gap generated force and 2 mm gap generated force in animal model, one author observed the gap, and the other author manually operated the machine. Any delay during this period might result in some deviation in the data. Sixth, the rat model is different from human tendons. We do not know if the current conclusion on animals is applicable to humans, so more clinical cases are needed to further confirm it.

Although our animal experiments may be limited to the laboratory scenario, the results of epidemiological analysis and animal experiments partly provided a certain degree of reference for predicting the probability of tendon injuries occurring by mechanical force at different ages and genders, as well as predicting the probability of good recovery after tendon injuries.

## Author Contributions

The study was conceptualized by Q.Q.Y. and Y.L.Z. Data was curated by Q.Q.Y. and Z.H. The funding was acquired by Q.Q.Y. and Y.L.Z. Experiments were performed by Q.Q.Y., Y.X., and J.Y.S. The methodology used was defined by Q.Q.Y., Z.H., Y.X., and J.Y.S. Z.H. and Y.L.Z. verified the underlying data. The original draft of this manuscript was written by Q.Q.Y. and edited by Y.L.Z. All the authors approved the final version of the manuscript.

## Funding

This work was supported by National Natural Science Foundation of China, 82372389. China Postdoctoral Science Foundation, 2025M772354. Nantong Natural Science Foundation, JC2025009.

## Conflicts of Interest

The authors declare no conflicts of interest.

## Supporting information


**Figure S1:** Temporal and spatial characteristics of disease burden. (A) Trends in the number of new cases and ASIR in China from 1990 to 2021. (B) Trends in the number of prevalent cases and ASPR in China from 1990 to 2021. (C) Relationship between ASIR and SDI in 21 regions. (D) Relationship between ASPR and SDI in 21 regions. (E) Relationship between ASIR and SDI across various countries. (F) Relationship between ASPR and SDI across various countries.

**Figure S2:** Differentially expressed genes of tendon cells with different genders by age. (A) Top 20 marker genes and (C) UMAP plot of major populations in tendon tissues. Clustering analysis revealed 4 distinct major cell populations containing fibroblasts, basal cells, epithelial cells, and immune cells. (B) Top 20 marker genes and (D) UMAP plot of subpopulations in tendon tissues. Clustering analysis revealed 20 subpopulations containing fibroblasts, chondrocytes, VSMCs, macrophages etc. Volcano diagram of differentially expressed genes among major cell populations containing (E) macrophages, DC cells, (F) endothelial cells and VSMCS. The red arrow represents the common differentially expressed genes (Col1a1 and Sparc) within the cell subpopulations.

**Figure S3:** Refined annotation of fibroblast subpopulations in the single‐cell RNA‐seq dataset. (A) UMAP visualization of re‐clustered fibroblast cells showing the original fibroblast subclusters. (B) UMAP visualization of fibroblast cells after refined annotation into tendon‐relevant stromal populations, including tenocyte‐like cells, epitenon‐like cells, ECM fibroblasts, repair fibroblasts, progenitor‐like fibroblasts, stress fibroblasts, and specialized fibroblasts. (C) DotPlot showing the expression of representative tendon stromal markers across the refined fibroblast subpopulations. (D) FeaturePlot showing the distribution of Creb3l1, Col1a1, and Sparc expression across fibroblast subpopulations. (E) Violin plots showing the expression of Creb3l1, Col1a1, and Sparc in tenocyte‐like cells across young and aged female and male groups.

**Figure S4:** Characteristics of tendon fibers with different genders by age. CellChat chord diagrams of cell interactions in (A) male rats and (B) female by age. H&E staining of tendon tissues in (C) male and (D) female rats. TEM images of tendon fibers in (E) male and (F) female rats.

**Figure S5:** Histological changes in repaired tendons. (A) Expression of Creb3l1, Col1a1 and Sparc proteins of repaired tendons in Con, LV‐NC and LV‐Creb3l1 groups for male and female rats after 3 weeks through immunohistochemical assay (*n* = 3 in each group). (B) H&E and Masson staining of repaired tendons in Con, LV‐NC and LV‐Creb3l1 groups for male and female rats after 3 weeks (*n* = 3 in each group).

**Figure S6:** Creb3l1 knockdown downregulates Col1a1 and Sparc and promotes tenocyte senescence. Relative mRNA levels of (A) *Creb3l1*, (B) *Col1a1* and (C) *Sparc*, and of the senescence markers (D) p16 and (E) p21, in tenocytes transfected with si‐NC or si‐Creb3l1. (F) Representative SA‐β‐gal staining (blue, senescent cells; scale bars, 200 μm) and (G) quantification of SA‐β‐gal–positive cells. Data are mean ± SD (*n* = 3); *p* values are indicated.

**Figure S7:** Macrophage polarization in repaired tendons.

**Table S1:** Literature‐guided comparison of tendon stromal populations identified in published mouse studies and the present rat single‐cell dataset. The table summarizes mouse tendon aging findings from Korcari et al. and epitenon‐related healing findings from Nichols et al., and compares these findings with the refined tenocyte‐like and epitenon‐like populations identified in the present rat dataset.

**Data S1:** acel70682‐sup‐0009‐FigureS1‐S7‐TableS1.docx.

## Data Availability

All data presented in this manuscript are available from the corresponding author Y.L.Z. (youlangzhou@163.com) upon request. The data related to mechanical‐force‐induced tendon injuries were visualized in RStudio, with the analysis integrating data from several public, freely accessible databases. GBD data are available from the Global Burden of Disease study (IHME) at https://www.healthdata.org/gbd and via the Global Health Data Exchange (GHDx) at https://ghdx.healthdata.org/gbd‐data. JASPAR database was used to predict potential Creb3l1‐binding sites in the *Col1a1* and *Sparc* promoters.
