# Supplementary material for: Creb3l1 Overexpression Improves Flexor Tendon Repair in Aged Rats
Source: Aging Cell. 2026 Aug 23;25(9):e70682. doi: 10.1111/acel.70682 (PMC13500903; doi:10.1111/acel.70682)
Supplement: Supplementary file 1 [file ACEL-25-e70682-s004.docx]

**Supplementary Table S1. Literature-guided comparison of tendon stromal populations in published mouse studies and the present rat single-cell dataset.**

| **Reference** | **Species / model** | **Tendon stromal population** | **Representative markers or defining features** | **Key published finding** | **Use in the revised manuscript** |
| --- | --- | --- | --- | --- | --- |
| Korcari et al., eLife 2023 | Mouse tendon aging | Tenocytes / tendon-lineage stromal cells | Tendon-lineage and matrix-producing cell states described by single-cell transcriptomic profiling. | The study reported age-associated remodeling of tendon stromal populations, including changes in matrix-producing tenocyte programs. | Provides the mouse aging context supporting our refined annotation of rat tenocyte-like cells rather than grouping all stromal cells as broad fibroblasts. |
| Korcari et al., eLife 2023 | Mouse tendon aging | ECM biosynthetic tenocyte-related programs | ECM-associated tendon programs and collagen/matrix-related transcriptional features. | Aging was associated with impaired ECM biosynthetic tendon cell programs in mouse tendon. | Consistent with our rat data showing reduced *Creb3l1*, *Col1a1*, and *Sparc* expression in aged tenocyte-like cells. |
| Nichols et al., Nature Communications 2025 | Mouse flexor tendon injury and healing | Epitenon-derived cells / epitenon-associated stromal cluster | Epitenon-associated stromal identity, including markers such as *Dpt*, *Col14a1*, *Ccn3*, and *Cd248* as reported in the healing context. | Epitenon-derived cells were shown to make distinct spatial and functional contributions to tendon healing and fibrosis. | Supports separate annotation of epitenon-like cells in our rat dataset rather than treating epitenon cells as generic fibroblast subclusters. |
| Present study | Rat tendon aging | Tenocyte-like cells | *Scx*, *Tnmd*, *Kera*, *Col1a1*, *Comp* | Tenocyte-like cells were resolved within the fibroblast/stromal compartment and showed age-associated reduction of *Creb3l1*, *Col1a1*, and *Sparc*. | Suggests that aging-related impairment of matrix-producing tendon stromal cells is at least partly conserved across rodent species. |
| Present study | Rat tendon aging | Epitenon-like cells | *Pi16*, *Col14a1*, *Dpt*, *C7* | Epitenon-like cells were identified as a distinct tendon stromal population after refined fibroblast annotation. | Aligns our rat single-cell annotation with emerging tendon literature emphasizing the distinct identity and function of epitenon cells. |
