# Supplementary material for: Creb3l1 Overexpression Improves Flexor Tendon Repair in Aged Rats
Source: Aging Cell. 2026 Aug 23;25(9):e70682. doi: 10.1111/acel.70682 (PMC13500903; doi:10.1111/acel.70682)
Supplement: Supplementary file 2 [file ACEL-25-e70682-s007.docx]

**Supplementary Figures**

**Supplementary Figure 1. Temporal and spatial characteristics of disease burden.** (A) Trends in the number of new cases and ASIR in China from 1990 to 2021. (B) Trends in the number of prevalent cases and ASPR in China from 1990 to 2021. (C) Relationship between ASIR and SDI in 21 regions. (D) Relationship between ASPR and SDI in 21 regions. (E) Relationship between ASIR and SDI across various countries. (F) Relationship between ASPR and SDI across various countries.

**Supplementary Figure 2. Differentially expressed genes of tendon cells with different genders by age.** (A) Top 20 marker genes and (C) UMAP plot of major populations in tendon tissues. Clustering analysis revealed 4 distinct major cell populations containing fibroblasts, basal cells, epithelial cells, and immune cells. (B) Top 20 marker genes and (D) UMAP plot of subpopulations in tendon tissues. Clustering analysis revealed 20 subpopulations containing fibroblasts, chondrocytes, VSMCs, macrophages etc. Volcano diagram of differentially expressed genes among major cell populations containing (E) macrophages, DC cells, (F) endothelial cells and VSMCS. The red arrow represents the common differentially expressed genes (Col1a1 and Sparc) within the cell subpopulations.

Supplementary Figure S3. Refined annotation of fibroblast subpopulations in the single-cell RNA-seq dataset. (A) UMAP visualization of re-clustered fibroblast cells showing the original fibroblast subclusters. (B) UMAP visualization of fibroblast cells after refined annotation into tendon-relevant stromal populations, including tenocyte-like cells, epitenon-like cells, ECM fibroblasts, repair fibroblasts, progenitor-like fibroblasts, stress fibroblasts, and specialized fibroblasts. (C) DotPlot showing the expression of representative tendon stromal markers across the refined fibroblast subpopulations. (D) FeaturePlot showing the distribution of Creb3l1, Col1a1, and Sparc expression across fibroblast subpopulations. (E) Violin plots showing the expression of Creb3l1, Col1a1, and Sparc in tenocyte-like cells across young and aged female and male groups.

**Supplementary Figure 4. Characteristics of tendon fibers with different genders by age.** CellChat chord diagrams of cell interactions in (A) male rats and (B) female by age. H&E staining of tendon tissues in (C) male and (D) female rats. TEM images of tendon fibers in (E) male and (F) female rats.

**Supplementary Figure 5. Histological changes in repaired tendons.** (A) Expression of Creb3l1, Col1a1 and Sparc proteins of repaired tendons in Con, LV-NC and LV-Creb3l1 groups for male and female rats after 3 weeks through immunohistochemical assay (n=3 in each group). (B) H&E and Masson staining of repaired tendons in Con, LV-NC and LV-Creb3l1 groups for male and female rats after 3 weeks (n=3 in each group).

**Supplementary Figure 6.** Creb3l1 knockdown downregulates Col1a1 and Sparc and promotes tenocyte senescence. Relative mRNA levels of (A) *Creb3l1*, (B) *Col1a1* and (C) *Sparc*, and of the senescence markers (D) P16 and (E) P21, in tenocytes transfected with si-NC or si-Creb3l1. (F) Representative SA-β-gal staining (blue, senescent cells; scale bars, 200 µm) and (G) quantification of SA-β-gal–positive cells. Data are mean ± SD (n = 3); P values are indicated.

**Supplementary Figure 7. Macrophage polarization in repaired tendons.**

| **Reference** | **Species / model** | **Tendon stromal population** | **Representative markers or defining features** | **Key published finding** | **Use in the revised manuscript** |
| --- | --- | --- | --- | --- | --- |
| Korcari et al., eLife 2023 | Mouse tendon aging | Tenocytes / tendon-lineage stromal cells | Tendon-lineage and matrix-producing cell states described by single-cell transcriptomic profiling. | The study reported age-associated remodeling of tendon stromal populations, including changes in matrix-producing tenocyte programs. | Provides the mouse aging context supporting our refined annotation of rat tenocyte-like cells rather than grouping all stromal cells as broad fibroblasts. |
| Korcari et al., eLife 2023 | Mouse tendon aging | ECM biosynthetic tenocyte-related programs | ECM-associated tendon programs and collagen/matrix-related transcriptional features. | Aging was associated with impaired ECM biosynthetic tendon cell programs in mouse tendon. | Consistent with our rat data showing reduced *Creb3l1*, *Col1a1*, and *Sparc* expression in aged tenocyte-like cells. |
| Nichols et al., Nature Communications 2025 | Mouse flexor tendon injury and healing | Epitenon-derived cells / epitenon-associated stromal cluster | Epitenon-associated stromal identity, including markers such as *Dpt*, *Col14a1*, *Ccn3*, and *Cd248* as reported in the healing context. | Epitenon-derived cells were shown to make distinct spatial and functional contributions to tendon healing and fibrosis. | Supports separate annotation of epitenon-like cells in our rat dataset rather than treating epitenon cells as generic fibroblast subclusters. |
| Present study | Rat tendon aging | Tenocyte-like cells | *Scx*, *Tnmd*, *Kera*, *Col1a1*, *Comp* | Tenocyte-like cells were resolved within the fibroblast/stromal compartment and showed age-associated reduction of *Creb3l1*, *Col1a1*, and *Sparc*. | Suggests that aging-related impairment of matrix-producing tendon stromal cells is at least partly conserved across rodent species. |
| Present study | Rat tendon aging | Epitenon-like cells | *Pi16*, *Col14a1*, *Dpt*, *C7* | Epitenon-like cells were identified as a distinct tendon stromal population after refined fibroblast annotation. | Aligns our rat single-cell annotation with emerging tendon literature emphasizing the distinct identity and function of epitenon cells. |

**Supplementary Table S1. Literature-guided comparison of tendon stromal populations identified in published mouse studies and the present rat single-cell dataset.** The table summarizes mouse tendon aging findings from Korcari et al. and epitenon-related healing findings from Nichols et al., and compares these findings with the refined tenocyte-like and epitenon-like populations identified in the present rat dataset.
